# Supplementary material for: Fear control and danger control amid COVID-19 dental crisis: Application of the Extended Parallel Process Model
Source: PLoS One. 2020 Aug 13;15(8):e0237490. doi: 10.1371/journal.pone.0237490 (PMC7425864; doi:10.1371/journal.pone.0237490)
Supplement: Attachment — Submitted filename: PONE-D-20-14093_reviewer.pdf [file pone.0237490.s001.pdf]

PLOS ONE

Fear Control and Danger Control Amid COVID-19 Dental Crisis: Application of the Extended Parallel Process Model

--Manuscript Draft--

**Manuscript Number:**	PONE-D-20-14093
**Article Type:**	Research Article
**Full Title:**	Fear Control and Danger Control Amid COVID-19 Dental Crisis: Application of the Extended Parallel Process Model
**Short Title:**	Fear Control and Danger Control Amid COVID-19 Dental Crisis
**Corresponding Author:**	Saeid Bashirian<br>Hamadan University of Medical Sciences School of Public Health<br>Hamedan, IRAN, ISLAMIC REPUBLIC OF
**Keywords:**	Fear control process; danger control process; Dentist; COVID 19
**Abstract:**	Objectives<br><br>There is high risk of contamination with COVID-19 virus during routine dental procedres and infection control is crucial. The aim of this study was to determine the factors associated with Covid-19 preventive behaviors among oral health care providers using an extended parallel process model (EPPM).<br><br>Methods<br><br>In a cross-sectional study, short text message invite surveys were sent to 870 oral health care providers in west part of Iran. Data were collected through validated self-report EPPM questionnaires. Descriptive statistics, Chi-square and Fishers exact tests were used for data analysis.<br><br>Results<br><br>In total, 300 completed questionnaires were received and the mean age of respondents was 29.89 ± 11.17 years (range: 20–75 years). Among the study population, 284 (94.67%) and 285 (95%) of participants had high perceived threat and high efficacy towards coronaviruse disease, respectively. The proportion of high efficacy was significantly higher in employees with more than 5-years of previous work history (P=0.04). washing hands frequently with water and soap/ use hand sanitizer was reported by 93.33%, of participants. Age (P=0.01), sex (P=0.002), previous work experience (P=0.05), and occupation field (P=0.01) were significantly associated with danger control responses. Data identified that those oral health care providers that were on the risk control response adopted preventive behaviors more strictly r than those on fear control response.<br><br>Conclusion<br><br>The results of this study showed how degrees of perceived threat and perceived efficacy influenced oral health providers' willingness to perform recommended health behaviors. These findings can assist public health agencies in developing educational programs specifically designed for promoting preventive behaviors among oral health providers in pandemic situations.
**Order of Authors:**	Samane Shirahmadi
	Shabnam Seyedzadeh-Sabounchi
	Salman Khazaei
	Saeid Bashirian
	Amir Farhang Miresmæili
	Zeinab Bayat

Powered by Editorial Manager® and ProduXion Manager® from Aries Systems Corporation

	Behzad Houshmand
	Hasan Semyari
	Majid Barati
	Ensiyeh Jenabi
	Fakhreddin Heidarian
	Sepideh Zareian
	Mohammad Kheirandish
	Neda Dadae
**Additional Information:**	
**Question**	**Response**
**Financial Disclosure**<br><br>Enter a financial disclosure statement that describes the sources of funding for the work included in this submission. Review the submission guidelines for detailed requirements. View published research articles from *PLOS ONE* for specific examples.<br><br>This statement is required for submission and **will appear in the published article** if the submission is accepted. Please make sure it is accurate.<br><br>**Unfunded studies**<br>Enter: *The author(s) received no specific funding for this work.*<br><br>**Funded studies**<br>Enter a statement with the following details:<br>• Initials of the authors who received each award<br>• Grant numbers awarded to each author<br>• The full name of each funder<br>• URL of each funder website<br>• Did the sponsors or funders play any role in the study design, data collection and analysis, decision to publish, or preparation of the manuscript?<br>• **NO** - Include this sentence at the end of your statement: *The funders had no role in study design, data collection and analysis, decision to publish, or preparation of the manuscript.*<br>• **YES** - Specify the role(s) played.<br><br>* typeset	The authors who received an award: Saeid Bashirian<br>Grant numbers awarded to author: IR.UMSHA.REC.1398.1094.<br>The full name of funder: the Vice Chancellor For research and technology from the Hamadan University of Medical Sciences.<br>The funders had no role in study design, data collection and analysis, decision to publish, or preparation of the manuscript.
**Competing Interests**	The authors have declared that no competing interests exist.

Use the instructions below to enter a competing interest statement for this submission. On behalf of all authors, disclose any competing interests that could be perceived to bias this work—acknowledging all financial support and any other relevant financial or non-financial competing interests.<br><br>This statement **will appear in the published article** if the submission is accepted. Please make sure it is accurate. View published research articles from *PLOS ONE* for specific examples.<br><br>**NO authors have competing interests**<br><br>Enter: *The authors have declared that no competing interests exist.*<br><br>**Authors with competing interests**<br><br>Enter competing interest details beginning with this statement:<br><br>*I have read the journal's policy and the authors of this manuscript have the following competing interests: [insert competing interests here]*<br><br>* typeset	
**Ethics Statement**<br><br>Enter an ethics statement for this submission. This statement is required if the study involved:<br><br>• Human participants<br>• Human specimens or tissue<br>• Vertebrate animals or cephalopods<br>• Vertebrate embryos or tissues<br>• Field research<br><br>Write "N/A" if the submission does not require an ethics statement.<br><br>General guidance is provided below.<br>Consult the submission guidelines for	The Ethics Committee of Hamadan University of Medical Sciences approved this study (IR.<br>UMSHA.REC.1398.1094). All data collected from the study participants were de-identified. No direct benefits or rewards were paid to participants for their participation in this study.

detailed instructions. **Make sure that all information entered here is included in the Methods section of the manuscript.**<br><br>**Format for specific study types**<br><br>**Human Subject Research (involving human participants and/or tissue)**<br>• Give the name of the institutional review board or ethics committee that approved the study<br>• Include the approval number and/or a statement indicating approval of this research<br>• Indicate the form of consent obtained (written/oral) or the reason that consent was not obtained (e.g. the data were analyzed anonymously)<br><br>**Animal Research (involving vertebrate animals, embryos or tissues)**<br>• Provide the name of the Institutional Animal Care and Use Committee (IACUC) or other relevant ethics board that reviewed the study protocol, and indicate whether they approved this research or granted a formal waiver of ethical approval<br>• Include an approval number if one was obtained<br>• If the study involved *non-human primates*, add *additional details* about animal welfare and steps taken to ameliorate suffering<br>• If anesthesia, euthanasia, or any kind of animal sacrifice is part of the study, include briefly which substances and/or methods were applied<br><br>**Field Research**<br><br>Include the following details if this study involves the collection of plant, animal, or other materials from a natural setting:<br>• Field permit number<br>• Name of the institution or relevant body that granted permission	
**Data Availability**<br><br>Authors are required to make all data underlying the findings described fully available, without restriction, and from the time of publication. PLOS allows rare	Yes - all data are fully available without restriction

exceptions to address legal and ethical concerns. See the PLOS Data Policy and FAQ for detailed information.<br><br>A Data Availability Statement describing where the data can be found is required at submission. Your answers to this question constitute the Data Availability Statement and **will be published in the article**, if accepted.<br><br>**Important:** Stating 'data available on request from the author' is not sufficient. If your data are only available upon request, select 'No' for the first question and explain your exceptional situation in the text box.<br><br>Do the authors confirm that all data underlying the findings described in their manuscript are fully available without restriction?	
**Describe where the data may be found in full sentences. If you are copying our sample text, replace any instances of XXX with the appropriate details.**<br><br>• If the data are **held or will be held in a public repository**, include URLs, accession numbers or DOIs. If this information will only be available after acceptance, indicate this by ticking the box below. For example: *All XXX files are available from the XXX database (accession number(s) XXX, XXX.).*<br>• If the data are all contained **within the manuscript and/or Supporting Information files**, enter the following: *All relevant data are within the manuscript and its Supporting Information files.*<br>• If neither of these applies but you are able to provide **details of access elsewhere**, with or without limitations, please do so. For example:<br><br>*Data cannot be shared publicly because of [XXX]. Data are available from the XXX Institutional Data Access / Ethics Committee (contact via XXX) for researchers who meet the criteria for*	All relevant data are within the manuscript.

access to confidential data.<br><br>*The data underlying the results presented in the study are available from (include the name of the third party and contact information or URL).*<br>• This text is appropriate if the data are owned by a third party and authors do not have permission to share the data.<br><br>* typeset	
Additional data availability information:	

Powered by Editorial Manager® and ProduXion Manager® from Aries Systems Corporation

Manuscript Click here to download Manuscript Main document.doc

Fear Control and Danger Control Amid COVID-19 Dental Crisis: Application of the Extended Parallel Process Model

Short title: Fear Control and Danger Control Amid COVID-19 Dental Crisis

Samane Shirahmadi[1], Shabnam Seyedzadeh-Sabounchi[1], Salman Khazaei[2], Saeid Bashirian[3*], Amir Farhang Miresmæili[4], Zeinab Bayat[5], Behzad Houshmand[6], Hasan Semyari[7], Majid Barati[3], Ensiyeh Jenabi[8], Fakhreddin Heidarian[9], Sepideh Zareian[10], Mohammad Kheirandish[11], Neda Dadae[11]

[1] Department of Community Oral Health, School of Dentistry and Dental Research Centers, Hamadan University of Medical Sciences, Hamadan, Iran.

[2] Assistant Professor of Epidemiology, Research Center for Health Sciences, Hamadan University of Medical Sciences, Hamadan, Iran.

[3] Associate Professor of Health Education and Promotion, Social Determinants of Health Research Center, Hamadan University of Medical Sciences, Hamadan, Iran.

[4] Department of Orthodontics, Dental Research Center, Hamadan University of Medical Sciences, Hamadan, Iran.

[5] Assistant Professor of Oral & Maxillofacial Medicine, Department of Oral Medicine, Hamadan University of Medical Sciences, Hmamedan, Iran.

[6] Professor of Periodontology, Department of Periodontics, School of Dentistry, Shahid Beheshti University of Medical Sciences, Tehran, Iran.

[7] Associate Professor of Periodontology, Department of Periodontics, School of Dentistry, Shahed University, Tehran, Iran.

[8] Assistant Professor of Mother and Child Health, Autism Spectrum Disorders Research Center, Hamadan University of Medical Sciences, Hamadan, Iran.

[9] DDS, PhD, Hamadan University of Medical Sciences, Hamadan, Iran.

[10] MSc of Computer Science, Hamadan University of Medical Sciences, Hamadan, Iran.

[11] Department Of Oral Health, Vice Chancellor For Health, Hamadan University of Medical Sciences, Hamadan, Iran.

*Corresponding Author: Saeid Bashirian, Email: bashirian@umsha.ac.ir

Abstract:

Objectives: There is high risk of contamination with COVID-19 virus during routine dental procedres and infection control is crucial. The aim of this study was to determine the factors associated with Covid-19 preventive behaviors among oral health care providers using an extended parallel process model (EPPM).

Methods: In a cross-sectional study, short text message invite surveys were sent to 870 oral health care providers in west part of Iran. Data were collected through validated self-report EPPM questionnaires. Descriptive statistics, Chi-square and Fishers exact tests were used for data analysis.

Results: In total, 300 completed questionnaires were received and the mean age of respondents was 29.89 ± 11.17 years (range: 20–75 years). Among the study population, 284 (94.67%) and 285 (95%) of participants had high perceived threat and high efficacy towards coronaviruse disease, respectively. The proportion of high efficacy was significantly higher in employees with more than 5-years of previous work history (P=0.04). washing hands frequently with water and soap/ use hand sanitizer was reported by 93.33%, of participants. Age (P=0.01), sex (P=0.002), previous work experience (P=0.05), and occupation field (P=0.01) were significantly associated with danger control responses. Data identified that those oral health care providers that were on the risk control response adopted preventive behaviors more strictly r than those on fear control response.

Conclusion: The results of this study showed how degrees of perceived threat and perceived efficacy influenced oral health providers' willingness to perform recommended health behaviors. These findings can assist public health agencies in developing educational programs specifically designed for promoting preventive behaviors among oral health providers in pandemic situations.

Key words: Fear control process, danger control process, Dentist, COVID 19

Interoduction:

Currently, the Coronavirus 2019 (COVID19) pandemic has become a major public health challenge (1). As of 28th of March, COVID19 has been diagnosed in 199 countries with 614,231 laboratory-confirmed cases and 28,240 deaths (2) .

Current observations suggest people of all ages are usually susceptible to this new viral disease. However, people who are in close contact with COVID-19 symptomatic and asymptomatic patients, including health care workers, are at higher risk for SARS-CoV-2 infection (3). Because healthcare workers are in direct contact with patients, they may be at risk when examining and treating patients. By not observaning to hygienic recommendations, healthcare professionals will become unwanted carriers and transmit the disease to other patients, their family members and to the community. Currently, one in five healthcare workers have been infected with COVID-19 (4).

Meanwhile, dentistry is one of the riskiest occupations. Since there is a high possibility of spread of and exposure to blood, body fluids, and respiratory secretions while providing dental services. Due to the characteristics of the dental settings, mutual infection between dentists and patients are possible. Therefore, the principle of infection control should be strictly enforced, to provide safety for both dentists and patients (5). Normal protective measures in daily clinical practice are not effective enough to prevent the spread of COVID-19 since a large quantity of droplets and suspended particles are created while removing decay or scaling and cleaning (3). Therefore, it is important to develop and follow accurate and effective preventive protocols. Dentists need to be encouraged to follow personal protection and infection control protocols carefully and thoroughly. In infectious disease epidemic campaigns, main focus is on motivating fear. However, one key factor in designing such educational programs is maximizing efficacy and avoiding unintended consequences such as fear and anxiety and disease-related stigma (6). Extended Parallel Processing Model

(EPPM) is one of the models that predicts the performance of health behaviors recommended by a person by analyzing fear appeals (7).

Due to the outbreak of coronavirus infection in Iran and the high importance of infection control in dentistry, the aim of this study was to determine the factors associated with Covid-19 preventive behaviors in oral health providers' using a model of EPPM. The results of this study can be used to design and implement appropriate educational interventions in order to prevent and control COVID-19 disease in dental settings.

Materials and Methods

Design

This cross-sectional study was conducted on March 2020 in the west part of Iran. In this study, factors affecting COVID-19 preventive behaviors in dentists was investigated using an Extended Parallel Processing Model.

Theoretical Framework

According to Extended Parallel Processing Model (EPPM), when people confront a serious threat, they are afraid and choose one of the two danger control or fear control responses to control the threat. When a person's motivation is danger control, he/she consciously thinks about the danger and ways to prevent the threat (protection motivation). In contrast, fear control processes focuses on controlling internal concerns (ie, emotions, physiological responses) (7).

The two main elements of danger perception in this model are perceived threat and perceived efficacy (Figure 1). Perceived threat consists of two basic dimensions: severity (susceptibility to the importance or magnitude of the threat) and sustainability (the person's belief in vulnerability to the threat). Perceived efficacy has two basic dimensions: response efficacy (effectiveness of the recommendations provided in preventing or counteracting the perceived

threat) and self-efficacy (a person's belief in his or her ability to follow advice) (7).

EPPM suggests that there is relationship between threat and multiplicative efficacy. So that in the conditions of high threat/high efficacy processes and consequences of danger control develop (ie, positive changes in attitudes, goals and behaviors) and high threat/low efficacy conditions initiate fear control processes and their outcomes (i.e., defensive avoidance or reactance).

Fig1. The Extended Parallel Process Model (EPPM) (7).

Sample and Setting:

In March 2020, a text message invitation to take a survey was sent to 860 dentists, faculty, and graduate or postgraduate dental students, dental assistants, and dental hygienists, in the city of Hamadan.

In total, 300 (34.8%) completed electronic questionnaires were received. The software application was designed in such a way that the oral health providers needed to answer all questions; otherwise, they did not receive a final confirmation.

The survey instrument:

The data collection instrument included a questionnaire consisting of two general sections of demographics and EPPM structures, which were completed by the participants in the study. Through the self-reporting method content validity of the EPPM scale was measured through content validity ratio (CVR) and content validity index (CVI). Eight health education, and promotion specialists and 2 dentists took part in the validation procedure. The CVR and CVI scores were 0.80 and 0.79, respectively. Face validity of the questionnaire was assessed by 30 dentists whose characteristics were similar to the target study sample.

The reliability of the questionnaire was assessed by calculating the internal reliability. Alpha Cronbach was 0.81 for "perceived sustainability", 0.78 for "perceived severity" 0.78, 0.84 for "self-efficacy", 0.87 for "response effectiveness" and 0.92 for "preventive behaviors."

Demographic variables included in the questionnaire were age, gender, previous work experience (year), occupation field (dentist, dental student, general dental student, Dental hygienist). The EPPM part of the questionnaire was consisted of 30 questions which covered five constrcuts described in Table 1.

Table 1. EPPM questionnaire about health behaviors regarding COVID-19 epidemic in oral health providers

Construct	questions (n)	Examples	Scale
**perceived severity**	3	Coronavirus can cause death	5-point Likert Scale from 1 (completely disagree) to 5 (completely agree)
**perceived susestbility**	2	I am unlikely to get coronavirus	5-point Likert Scale from 1 (completely disagree) to 5 (completely agree)
**response efficacy**	11	Wearing goggles, masks or shields during all stages of treatment is effective in preventing coronavirus.	5-point Likert Scale from 1 (completely disagree) to 5 (completely agree)
**self-efficacy**	7	I can use goggles, masks or shields during all stages of treatment	5-point Likert Scale from 1 (completely disagree) to 5 (completely agree)
**preventive behavior**	7	I wear goggles and shield masks during all stages of treatment	1-3 Likert scale which 1 represented never and 3 was always

Data Analysis:

The statistical analysis was performed using SPSS version 16.0 software. Using descriptive statistics (mean, standard deviation, frequency and percentage) in the form of tables and graphs, the study population was described. The Chi-Square test and Fisher Accurate tests were used to compare the level of threat and perceived efficiency based on demographic variables.

Using Likert scale responses, the "threat" constrcut was developed as a product of participants' response to perceived sustainability and perceived severity. The "efficacy" construct was identified through calculating the participants' responses to self-efficacy and efficacy.

Witte discriminating value was used to measure danger control responses (8) and with Chi-square test, the comparison of demographic variables was performed at the levels formed by the model (danger control and fear control response).

Low and high values of threat and perceived efficacy were determined by the median value of each structure, respectively. The four EPPM classifications were created based on the perceived threat level and the perceived efficacy level. These classes included low threat and efficacy (LT/LE), low threat and high efficacy (LT/HE), high threat and low efficacy (HT/LE), and finally high threat and efficacy (HT/HE).

Using the Fisher Accurate Test, the performance of the seven preventive behaviors examined at the levels formed by the model (LT/LE, HT/LE, LT/HE, HT/HE) were compared. Furthermore, 7 preventive behaviors were compared by using Chi-square test according to the levels formed by the model (danger control and fear control paths). We reduced the preventive behavior responses to always and sometimes since the number of never responses were low. Significance level in all tests was considered at $p<0.05$.

Ethical Consideration

The Ethics Committee of Hamadan University of Medical Sciences approved this study (IR. UMSHA.REC.1398.1094). All data collected from the study participants were de-identified. No direct benefits or rewards were paid to participants for their participation in this study.

Results

In total, 300 participations with the mean age of 29.89 ± 11.17 years (range: 20–75 years) were investigated. There were 160 (53.33%) male participants and 212 (70.67%) had previous work experience of less than 5 years. Based on occupation field , 175 (58.63%) were dentists and 98 (32.67%) were dental students (Table 2).

In table 2, associations between respondents' demographic characteristics and perceived threat and efficacy to COVID-19 are demonstarted. In this regard, 284 (94.67%) and 285 (95%) of the study populations had high perceived threat and efficacy, respectively. The proportion of high efficacy was significantly higher is employees with more than 5-years of previous work experience (P=0.04) and those older than 30 years of age (P=0.08). There was not a significant different between respondents' demographic characteristics and their perceived threat (P>0.05).

Table 2. Associations between respondents' demographic characteristics and perceived threat and efficacy to COVID-19 epidemic

Variable		Total N (%)	Threat		P_{Value}	Efficacy		P_{Value}
			Low N (%)	High N (%)		Low N (%)	High N (%)	
Gender	Male	160 (53.3)	10(6.2)	150 (93.7)	0.45	6(3.7)	154(96.2)	0.29
	Female	140(46.6)	6(4.2)	134 (95.7)		9(6.4)	131(93.5)	
Age group (year)	20-29	211 (70.3)	12(5.6)	199(94.3)	0.89	15(7.1)	196(92.8)	0.08
	30-39	37(12.3)	1(2.7)	36(97.3)		0	37(100.0)	
	40-49	14(4.6)	1(7.1)	13(92.8)		0	14(100.0)	
	50-60	38(12.6)	2(5.2)	36(94.7)		0	38(100.0)	
Previous work experience (year)	<5	212(70.6)	12(5.6)	200(94.3)	0.59	15(7.0)	197(92.9)	**0.04**
	5-10	44(14.6)	1(2.2)	43(97.7)		0	44(100.0)	
	>10	44(14.6)	3(6.8)	41(9.1)		0	44(100.0)	
Occupation Field	Dentist	175(58.3)	6(3.4)	169(96.5)	0.22	6(3.4)	169(96.5)	0.22
	Student	98(32.6)	8(8.1)	90(91.8)		8(8.1)	90(91.8)	
	Dental hygienist	27(9.0)	2(7.4)	25(92.5)		1(3.7)	26(96.3)	
Total		300(100.0)	16(5.3)	284(94.6)	-	15(5.0)	285(95.0)	-

preventive behaviors of study participations regarding prevention of COVID-19 are presented in figure 2. Approximately 93.33% of the partcipants washed their hands with water and soap frequently or used hand disinfectant.

Fig 2. Health behaviors of oral health provider regarding prevention of COVID-19

(Behavior 1: washing hands frequently with water and soap/ use hand sanitizer, behavior 2: Do not admit suspicious patients in the Dental office, behavior 3: Avoid close contact, behavior 4: use goggles, masks or shields during all treatment steps, behavior 5: use protective uniforms (gowns , apron, protective gloves) during all treatment steps, behavior 6:

Disinfect equipment and surfaces regularly, behavior 7: Request patients to wash their hands with soap and water upon entering the dental clinic.

In accordance with the EPPM model, the proportion of participations with low perceived threat and efficacy (LT/LE), low threat-high efficacy(LT/HE), High threat-low efficacy (HT/LE) and high both perceived threat and efficacy (HT/HE) were 0.67%, 4.67%, 4.33% and 90.33%, respectively (Table 3).

Preventive behaviors regarding COVID-19 epidemic in oral health providers based on different levels of the EPPM model is shown in table 3. Oral health providers with high threat-high efficacy significantly performed behavior 6 (disinfect equipment and surfaces regularly) more than other categories (P=0.055). In relation with other behaviors there were no significant differences between EPPM categories.

Table 3.Health behaviors regarding COVID-19 epidemic in oral health providers in different levels of the EPPM

**Behavior**		**Low threat-Low efficacy N (%)**	**Low threat-High efficacy N (%)**	**High threat-Low efficacy N (%)**	**High threat-High efficacy N (%)**	**P.Value**
Behavior 1	Some times	0	1 (7.1)	1 (7.6)	18 (6.6)	0.87
	Always	2 (100.0)	13 (92.8)	12 (92.3)	253 (93.3)	
Behavior 2	Some times	1 (50.0)	7 (50.0)	3 (23.0)	83 (30.6)	0.32
	Always	1 (50.0)	7 (50.0)	10 (76.9)	188 (69.3)	
Behavior 3	Some times	0	2 (14.2)	1 (7.6)	19 (7.0)	0.54
	Always	2 (100.0)	12 (85.7)	12 (92.3)	252 (92.9)	
Behavior 4	Some times	0	3 (21.4)	3 (23.0)	41 (15.1)	0.40
	Always	2 (100.0)	11 (78.5)	10 (76.9)	230 (84.8)	
Behavior 5	Some times	0	2 (14.2)	4 (30.7)	81 (29.8)	0.58
	Always	2 (100.0)	12 (85.7)	9 (69.2)	190 (70.1)	
Behavior 6	Some times	0	3 (21.4)	5 (38.4)	35 (12.9)	**0.055**
	Always	2 (100.0)	11 (78.5)	8 (61.5)	236 (87.0)	
Behavior 7	Some times	0	5 (35.7)	5 (38.4)	92 (33.9)	0.89
	Always	2 (100.0)	9 (64.2)	8 (61.5)	179 (66.0)	
Total		2 (0.67)	14 (4.67)	13 (4.33)	271 (90.33)	

Behavior 1: washing frequently hands with water and soap and a hygienic hand disinfection, behavior2: Do not visit the patient or suspicious people in the Dental office, behavior 3: Avoid close contact, behavior 4: use of goggles, masks or shields during all treatments, behavior 5: use of protective uniforms (gowns , apron, protective gloves) during all treatment steps, behavior 6: Disinfect equipment and surfaces, behavior 7: Requiring patients to wash their hands with soap and water upon entering the Dental clinic.

The numbers in Figure 3 shows that 58.5% of women, 45.0% of the 20-29 age group, 43.4% of participants with less than 5 years of previous work experience, and 62.9% of dental hygienists were in the fear control process. Furthermore, age (P=0.01), sex (P=0.002), previous work experience (P=0.05) and field of occupation (P= 0.01) were significantly associated with the danger control response.

Fig 3. Results of the discriminating value formula for danger control responses

Discussion

The results of the present study identified that the perceived threat and efficacy construct among the study participants were at a high level (94.6% high perceived threat and 95% high perceived efficiency). Females, younger participants, those with less previous work experience and dental hygienists were more motivated to control their fears than other groups.

The results of this study showed that previous work experiencehas a significant relationship with the perceived efficacy. Participants with higher previous work experiencewere more efficient. Similar results have been reported by Wilson (9) and Raybould (10). The most important influencing source on efficacy is past experience according to the literature (11). Therefore, previous experience of dealing with various epidemics, including influenza and infectious diseases such as AIDS and hepatitis, can increase the efficiency of providers with longer previous work experience (12).

The trends observed in previous studies (13, 14) and the results of this study show that the efficacy dimension of EPPM has a more positive effect on health behavior than the threat dimension. This pattern was also observed in preventive behaviors numbers 4 to 7 (use goggles, masks or shields during all treatment steps; wear protective uniforms (gowns, aprons, protective gloves during all treatment steps; disinfect equipment and surfaces and regularly; request patients to wash their hands with soap and water upon entering the Dental

office). This indicates a more positive effect of the perceived effecacy dimension than the perceived threat dimension on the recommended health behaviors. Therefore, it seems that in viral epidemics, in order to intervene and provide guidance, more emphasis should be placed on the efficiency dimension.

The centers for disease control and prevention (CDC) the national association of dentists strongly advise dentists to discontinue unnecessary dental treatments during the epidemic period and not to treat or examine people suspected of having the disease in regular dental clinics (15, 16). The results of this study show that although 90.3% of the participants in the study were in the HTHE class, only 69.3% of them avoided to visit people with suspected disease in their office. The reason for this avoidance can be that they had high perceived response efficacy since 95% of the participants considered the recommended health behaviors to be effective in preventing the transmission of the disease and perceived their ability as high to perform the preventive behaviors.

In this study, the calculated discriminating value determined whether participants were motivated to control fear or to control danger similar to previous studies (8). The results identified a statistically significant difference between the different groups studied in terms of fear control and danger control. This shows that the members of different occupation field groups respond differently when faced with a health problem, such as COVID 19. Therefore, differences between groups should be taken into consideration while designing interventions based on groups' specific characteristics and conditions.

This study has several important strengths. First, COVID 19 infection had been selected since it is currently an international public health problem. Second, given that the health messages distributed in this pandemic situation has not successfully influenced perceptions, this study determined the control processes. Based on the results, specific target groups can be identified and the necessary interventions can be tailored according to the control processes.

One of the limitations of this study is the bias induced by social utility. Although, we tried to minimize the influence of social utility while constructing and expressing the content of the questionnaire, this survey-based study does not necessarily predict actual behavior during an event. Also, the findings of our study on oral health care providers may not necessarily lead to similar findings among other health care providers such as hospital staff and nurses.

Cross-sectional studies do not determine the causal relationship between the recommended health behaviors and the levels formed by the model (danger control and fear control). However, cross-sectional studies are an important tool for identifying danger and protection indicators for future longitudinal assessments.

Conclusion

The results of this study showed how degrees of perceived threat and perceived efficacy affect dentists' willingness to perform recommended health behaviors. Women's danger control was lower than that of men. Dental students and dental hygenists were less motivated to control danger.

Given that the coronavirus pandemic is an important public health issue, and the protection of medical staff such as dentists and patient safety in clinical care should be taken seriously, the placement of oral health care provider in the motivation of fear control, will lead to neglect of key health messages and actions or relevant behaviors to prevent COVID-19. Therefore, it is necesssary to develop educational programs to increase the efficacy of oral health professionals in helping to prevent the spread of the disease.

Acknowledgements

This work was funded by a grant from the Vice Chancellor For research and technology (IR.UMSHA.REC.1398.1094) from the Hamadan University of Medical Sciences. The authors declare that they have no competing interests.

Conflict OF Interests

The authors have no conflict of interests.

Authors' Contributions

Conceptualization: Samane Shirahmadi, Saeid Bashirian.

Data Curation: Samane Shirahmadi, Salman Khazaei.

Formal Analysis: Salman Khazaei, Samane Shirahmadi, Saeid Bashirian.

Funding Acquisition: Saeid Bashirian.

Investigation: Samane Shirahmadi, Amir Farhang Miresmæili, Shabnam Seyedzadeh-Sabounchi, Zeinab Bayat, Behzad Houshmand, Hasan Semyari, Majid Barati, Ensiyeh Jenabi, Fakhreddin Heidarian.

Methodology: Saeid Bashirian, Samane Shirahmadi.

Project Administration: Saeid Bashirian, Samane Shirahmadi.

Resources: Fakhreddin Heidarian**,** Mohammad Kheirandish, Neda Dadae.

Software: Salman Khazaei, Samane Shirahmadi, Sepideh Zareian.

Supervision: Saeid Bashirian, Samane Shirahmadi.

Validation: Amir Farhang Miresmæili, Shabnam Seyedzadeh-Sabounchi, Zeinab Bayat, Behzad Houshmand, Hasan Semyari, Majid Barati.

Writing– Original Draft: Samane Shirahmadi, Shabnam Seyedzadeh-Sabounchi, Salman Khazaei, Saeid Bashirian.

Review&Editing: Samane Shirahmadi, Shabnam Seyedzadeh-Sabounchi, Salman Khazaei, Saeid Bashirian, Amir Farhang Miresmæili, Zeinab Bayat, Behzad Houshmand, Hasan

Semyari, Majid Barati, Ensiyeh Jenabi, Fakhreddin Heidarian, Sepideh Zareian, Mohammad Kheirandish, Neda Dadae.

References

1. Phelan AL, Katz R, Gostin LO. The novel coronavirus originating in Wuhan, China: challenges for global health governance. Jama. 2020;323(8):709-10.

2. Organization WH. Coronavirus disease 2019 (COVID-19): situation report, 67. 2020
.

3. Meng L, Hua F ,Bian Z. Coronavirus Disease 2019 (COVID-19): Emerging and Future Challenges for Dental and Oral Medicine. Journal of Dental Research. 2020;99(5):481-7.

4. Wang C, Horby PW, Hayden FG, Gao GF. A novel coronavirus outbreak of global health concern. The Lancet. 2020;395(10223):470-3.

5. DePaola LG, Grant LE. Summary of Infection Control in the Dental Office: A Global Prospective. Infection Control in the Dental Office: Springer; 2020. p. 213-6.

6. Perloff RM. The dynamics of persuasion: communication and attitudes in the twenty-first century: Routledge; 2010.

7. Witte K. Fear control and danger control: A test of the extended parallel process model (EPPM). Communications Monographs. 1994;61(2):113-34.

8. Witte K. Predicting risk behaviors: Development and validation of a diagnostic scale. Journal of health communication. 1996;1(4):317-42.

9. Wilson M, Byers T. Relationship Between Self-Efficacy and Work Experience in Baccalaureate Junior and Senior Level Nursing Students. Honors Research Projects. 2017;42.

10. Raybould M, Pratt M, Walters G, editors. The effects of work experience on university students' self-efficacy related to employment and study related skills. CAUTHE 2016–26th CAUTHE conference: The Changing Landscape: The Impact of Emerging Markets and Destinations; 2016: Blue Mountains International Hotel Management School.

11. Bandura A. Perceived self-efficacy in cognitive development and functioning. Educational psychologist. 1993;28(2):117-48.

12. Zamani-Alavijeh F, Araban M, Harandy TF, Bastami F, Almasian M. Sources of Health care providers’ Self-efficacy to deliver Health Education: a qualitative study. BMC medical education. 2019;19(1):1-9.

13. Barnett DJ, Balicer RD, Thompson CB, Storey JD, Omer SB, Semon NL, et al. Assessment of local public health workers' willingness to respond to pandemic influenza through application of the extended parallel process model. PloS one. 2009;4(7):1-8.

14. Roberto AJ, Mongeau PA, Liu Y, Hashi EC. “Fear the Flu, Not the Flu Shot”: A Test of the Extended Parallel Process Model. Journal of health communication. 2019;24(11):829-36

15. Prevention CfDCa. Interim Infection Prevention and Control Recommendations for Patients with Suspected or Confirmed Coronavirus Disease 2019 (COVID-19) in Healthcare Settings :Centers for Disease Control and Prevention. Available from: https://www.cdc.gov/coronavirus/2019-ncov/hcp/infection-control recommendations.html?CDC_AA_refVal=https%3A%2F%2Fwww.cdc.gov%2Fcoronavirus%2F2019 ncov%2Finfection-control%2Fcontrol-recommendations.html.

16. Prevention CfDCa. Interim Infection Prevention and Control Guidance for Dental Settings During the COVID-19 Response 2020. Available from: https://www.cdc.gov/coronavirus/2019-ncov/hcp/dental-settings.html.

 Click here to download Figure Fig 1.tif

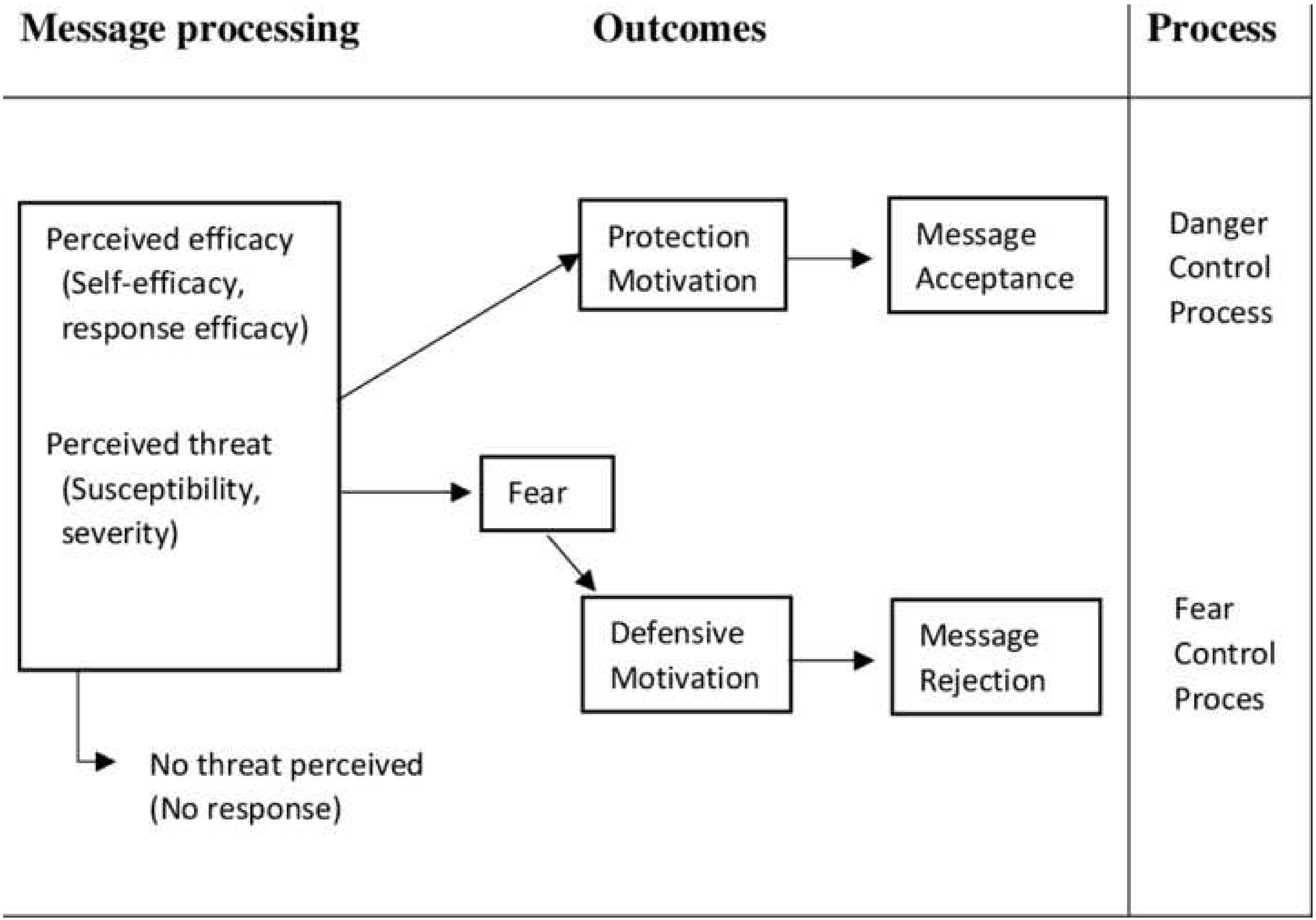

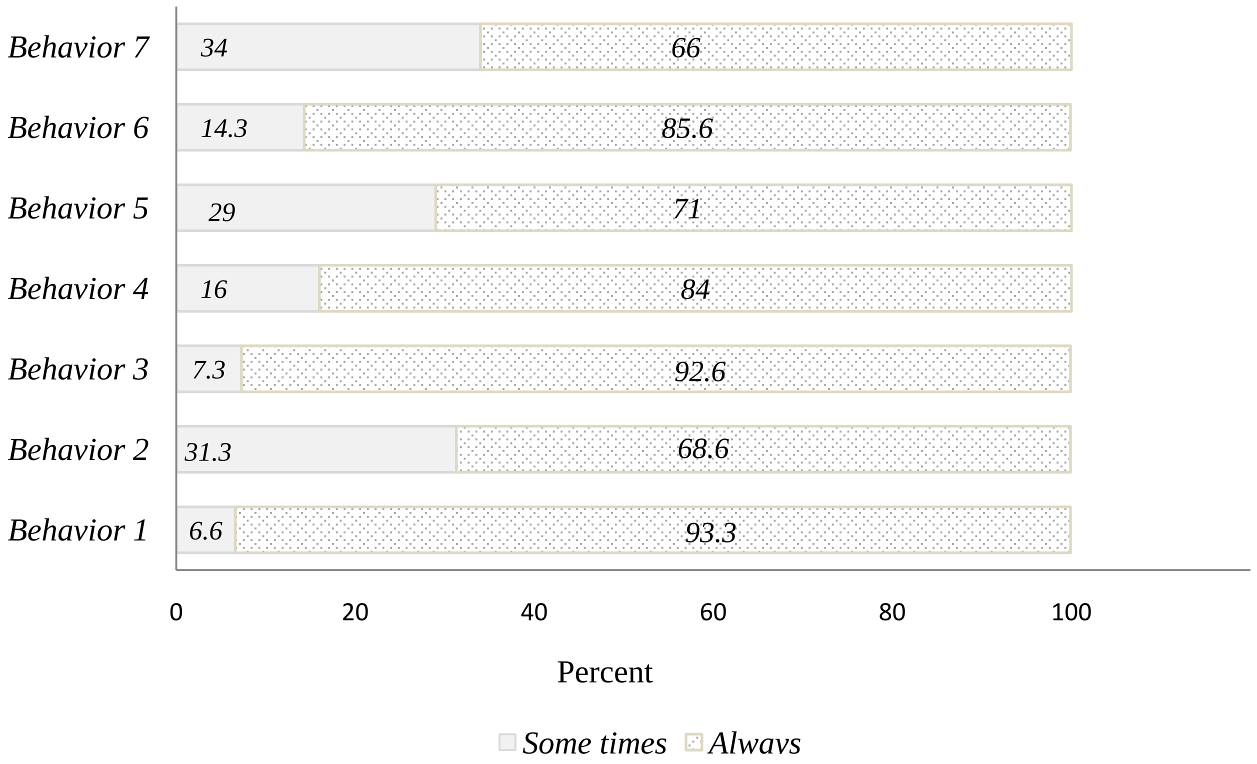

Fig 2. Health behaviors of oral health provider regarding prevention of COVID-19

Figure Click here to download Figure Fig 3.pdf

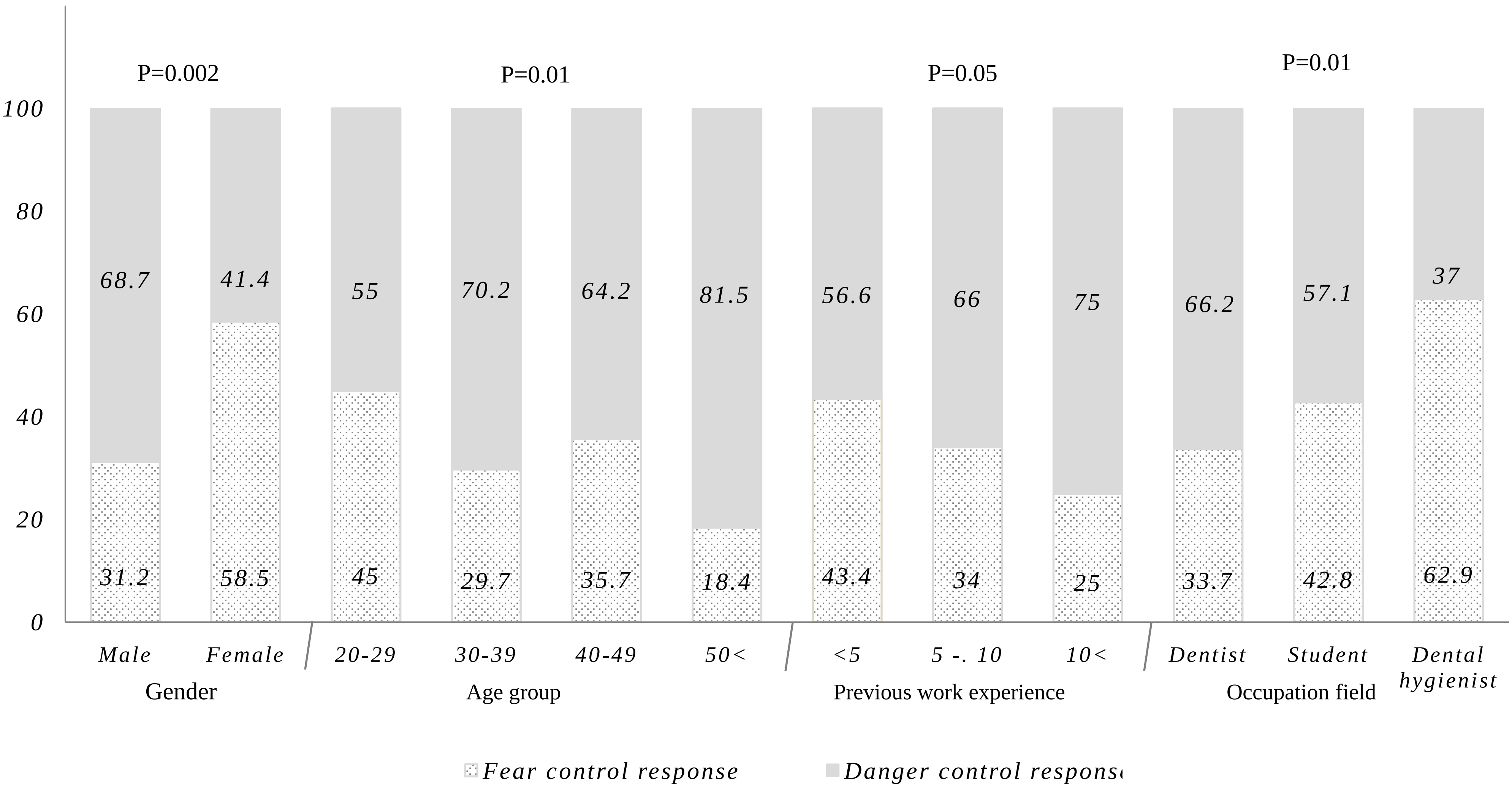

Fig3. Results of the discriminating value formula for danger control responses
