# Supplementary material for: Fear control and danger control amid COVID-19 dental crisis: Application of the Extended Parallel Process Model
Source: PLoS One. 2020 Aug 13;15(8):e0237490. doi: 10.1371/journal.pone.0237490 (PMC7425864; doi:10.1371/journal.pone.0237490)
Supplement: Attachment — Submitted filename: Response to Reviewers.doc [file pone.0237490.s003.doc]

*Author’s response to reviews*

**Title: Fear Control and Danger Control Amid COVID-19 Dental Crisis: Application of the Extended Parallel Process Model**

**Authors:**

Samaneh Shirahmadi ([shirahmadi_s@yahoo.com](mailto:shirahmadi_s@yahoo.com))

Shabnam Seyedzadeh-Sabounchi,

Salman Khazaei,

Saeid Bashirian,

Amir Farhang Miresmæili,

Zeinab Bayat,

Behzad Houshmand

Hasan Semyari

Majid Barati,

Ensiyeh Jenabi,

Fakhreddin Heidarian,

Sepideh Zareian,

Mohammad Kheirandish,

Neda Dadae

**Version:** 2	**Date:** 25 July 2020

**Author's response to reviews:** see over

We thank all the Reviewers for their valuable feedback and taking the time to provide useful comments to improve our manuscript entitled “Fear Control and Danger Control amid COVID-19 Dental Crisis: Application of the Extended Parallel Process Model”.

Based on the constructive comments the following changes have been made.

**Response to Reviewer 3:**

1. Please note that one/two sentences do not constitute one paragraph. Please revise carefully, and summarize your thoughts in reasonable sections. Revise throughout your text.

Response: Thank you for your comments. We have incorporated this advice in all sections of the manuscript and revised accordingly.

2. "As of 28th of March, COVID19 has been diagnosed in 199 countries with 614,231 laboratory-confirmed cases and 28,240 deaths (2)." Please update your information. Meanwhile, it is July.

Response: Thank you for your valuable comment. The sentence has been revised as: "As of 20^th^ of July, COVID19 has been diagnosed in 213 countries with 14,855,107 laboratory-confirmed cases and 613,248 deaths"***.*** Page 3, Line 70.

And "revised these sentences "As of 25^th^ of March 2020, COVID 19 had been diagnosed in Iran with 27017 laboratory-confirmed cases and 2077 deaths had been recorded": As of 21^st^ of July 2020, COVID 19 had been diagnosed in Iran with 276,202 laboratory-confirmed cases and 14,405 deaths had been recorded." Page 13, Line 300.

3. Revise thoroughly for spacebar use.

Response: Thank you for your comments. We have revised the manuscript thoroughly for the proper use of spacebar.

4. "(...)COVID-19 epidemic.” "Please revise for quotation marks not considered necessary.

Response: Thank you for your comments. We have revised and removed unnecessary quotation marks.

5. p≤0.05 must read p<0.05.

Response: Significance level in all tests was considered at p<0.05." Page 8, Line 202.

6. All p values must be given on a three-digit basis. Revise thoroughly.

Response: Thank you for your comments. We have revised all p values***.***

7. Again, please revise carefully. "efficacy(LT/HE)" should read "efficacy (LT/HE)".

Response: Thank you for your comments***.*** We have revised our manuscript.

8. Same with "Table 3. Health (...)".

Response: Thank you for your comments***.*** We have revised our manuscript.

9. Same with "P.Value". Delete your dot.

Response: Thank you for your comments***.*** We have revised all p values***.***

10. Please compare: behavior2:, behavior 4:, "(gowns , apron, (...)".

Response: Thank you for your valuable comment. These two behaviors reflect two different behaviors of protecting the eyes and the other is about wearing gloves and protective clothing which we have revised in the manuscript to clearly state these two different behaviors for the readers " use protection for eyes such as goggles, masks or shields during all treatments and use of protective uniforms (gown or protective clothing, protective gloves) during all treatment steps

11. Please revise: "disease -related guidelines", "self-regulation model, etc.," What is "etc"? Why closing this sentence with a comma?

Response: Thank you for your comments***.*** The sentence has been revised as:" These interventions include educating providers on corona virus disease-related guidelines and recommendations for conducting health behaviors using health education models. The focus of the models can be directed towards increasing self-efficacy, such as self-efficacy model, self-regulation model and using implementation intention theory. Page 14, Line 323.

12. Referees still not uniformly formatted. Please see Authors' Guidelines.

Response: Thank you for your comments***.*** We have revised all references***.***
